# Energy profile and secondary structure impact shRNA efficacy

**DOI:** 10.1186/1471-2164-10-S1-S9

**Published:** 2009-07-07

**Authors:** Hong Zhou, Xiao Zeng

**Affiliations:** 1 Saint Joseph College, 1678 Asylum Avenue, West Hartford, CT 06117, USA; 2 SABiosciences Corporation, 6951 Executive Way, Frederick, MD 21703, USA

## Abstract

**Background:**

RNA interference (RNAi) is a cellular mechanism in which a short/small double stranded RNA induces the degradation of its sequence specific target mRNA, leading to specific gene silencing. Since its discovery, RNAi has become a powerful biological technique for gene function studies and drug discovery. The very first requirement of applying RNAi is to design functional small interfering RNA (siRNA) that can uniquely induce the degradation of the targeted mRNA. It has been shown that many functional synthetic siRNAs share some common characteristics, such as GC content limitation and free energy preferences at both terminals, etc.

**Results:**

Our three-phase algorithm was developed to design siRNA on a whole-genome scale based on those identified characteristics of functional siRNA. When this algorithm was applied to design short hairpin RNA (shRNA), the validated success rate of shRNAs was over 70%, which was almost double the rate reported for TRC library. This indicates that the designs of siRNA and shRNA may share the same concerns. Further analysis of the shRNA dataset of 444 designs reveals that the high free energy states of the two terminals have the largest positive impact on the shRNA efficacy. Enforcing these energy characteristics of both terminals can further improve the shRNA design success rate to 83.1%. We also found that functional shRNAs have less probability for their 3' terminals to be involved in mRNA secondary structure formation.

**Conclusion:**

Functional shRNAs prefer high free energy states at both terminals. High free energy states of the two terminals were found to be the largest positive impact factor on shRNA efficacy. In addition, the accessibility of the 3' terminal is another key factor to shRNA efficacy.

## Background

RNA interference (RNAi) is a cellular mechanism in which a short/small double stranded RNA induces the degradation of its sequence specific target mRNA, leading to specific gene silencing. Since its discovery, RNAi has become a powerful biological technique for gene function studies and drug discovery [1-3]. It also shows promise as a direct therapeutic agent [4-6]. In order to apply RNAi technology, one must scan the target sequence and identify a stretch of 19 ~29 nucleotide sequence that might give the best chance to succeed as gene-specific small interfering RNA (siRNA) because randomly selected siRNA is seldom functional [7]. Previous research has identified some empirical rules regarding the efficacy of siRNA sequences. These rules include the GC content limitation (the optimal GC content is between 30% and 55%) [7-13], the thermo-stability preference (lower stability at the 3' terminal of sense strand would help the antisense strand enter into the RISC complex) [11,14,15], and some base preferences in the siRNA sequences [7,16]. Recently, several studies employed computational methods to analyze the published functional siRNA datasets whose sizes are relatively large and revealed more base preferences at particular positions [17-20]. These computational models and their discoveries seem to be promising, because they were using large set of experimentally validated sequences. However, one must be cautious when reading into these conclusions because the existing datasets might be biased for lack of negative results (some negative results were seldom reported in publications).

Another challenge is that most existing datasets are usually about chemically synthesized siRNA sequences. Currently there are two approaches to induce siRNA sequences into cells. One is to transfect chemically synthesized siRNA sequences into cells. Though this approach is more frequently used, the drawback is that it can not offer long-term gene suppression and some mammalian cell types are resistant to the transfection methods [21,22]. The alternative approach is to have a small hairpin RNA or short hairpin RNA (shRNA) expressed on a plasmid vector that enables long-term gene suppression [22]. Another advantage of using the shRNA approach is that once a functional vector is identified, it can be reproduced easily and inexpensively.

As suggested by Matveeva et al [19], some of the characteristics of functional siRNA may not apply to shRNA, though many of them may. In 2004 and 2005, we developed a three-phase algorithm for computer-aided siRNA design [23,24]. This algorithm includes all the design considerations published by then and arranges all the necessary siRNA selection rules in three groups of filters according to their impacts on the siRNA efficacy and applies them to the design process in three steps. Each filter represents a specific design rule. Phase I filters eliminate all the siRNA sequences containing the damaging elements for a functional siRNA. Examples are those filters that prohibit the existence of internal palindromes or long GC stretches. All siRNA candidates must successfully pass all the phase I filter assessment. Phase II filters are used to rank eligible siRNA sequences by a final score with the sum of gain and penalty points. There is a cutoff such that siRNA candidates will be selected only if their final scores are no less than the cutoff point. Phase III filters represent those rules whose impact on the siRNA functionality has yet to be clarified and therefore are considered optional. Most of the selective filters in Phase II are set to ensure the selection rule that the 3' terminal is less thermodynamically stable, compared to the 5' end (on the sense strand). This differential stability increases the probability that the antisense strand will be incorporated into the RISC complex [14,15]. This algorithm was adopted by SABiosciences Corporation and has been eventually used for their shRNA design. Biological experiments conducted by SABiosciences have shown that 71.2% (316/444) of the shRNAs designed by our algorithm are functional (i.e. can suppress at least 70% of the target gene expression in average), a high success rate that has never been reported experimentally with shRNA (for the definition of a functional shRNA or a successful design of shRNA, please read the section Methods). For example, the design algorithms employed recently by Root [22] and Moffat [21] for shRNA generate less than 40% functional shRNAs. Since our algorithm was originally developed based on discoveries and studies concerning siRNA functionality, its high success rate in shRNA design suggests that most design concerns for siRNA sequences are applicable to shRNA design.

This article further extends our analysis on the available shRNA dataset generated by SABiosciences. Although this dataset is biased as it is specifically generated by our three-phase algorithm, the analysis reveals useful information that may help confirm the effectiveness of the rules used in the algorithm, modify the existing rules or rearrange them for better prediction and identify new rules.

## Results and discussion

The two sets of biological experiments completely tested 444 shRNAs targeting 125 human genes. Of the 444 shRNAs, 316 are found to be functional (71.2%). Considering the fact that variations exist in the experimentally measured suppression efficacy, we decided to remove some shRNAs whose efficacy are in the range near 70% in hope of ensuring the validity of the dataset. The two ranges for removal are 60–75% and 55–80%. This means we exclude shRNAs whose efficacy is between 60–75% and 55–80% respectively. With the 60–75% removal range, there are 351 shRNAs for analysis in which 268 are considered functional (efficacy >= 75%). However, with the 55–80% range, there are only 289 shRNA sequences for analysis in which 221 shRNAs are considered functional (efficacy >= 80%).

By default, the three-phase algorithm sets a selection cutoff such that only shRNAs which score at least 7 points will be selected for biological experiments. However, former experiments showed that there were a few genes for which our algorithm failed to design enough shRNAs [23]. Thus in the dataset there are some shRNAs scoring less than 7 points. For this reason, the chi-square test for independence, is first applied to assess if the 7-points-cutoff is significant in distinguishing between functional and nonfunctional shRNAs. Note that all further statistical results are obtained using the chi-square test unless otherwise noted. The analysis shows that the 7-points-cutoff is significant in making the distinction between the functional and nonfunctional shRNAs. shRNAs scoring at least 7 points have a higher probability of being functional. When the removal range is 60–75%, the p-value is 0.037. With the removal range to be 55–80%, the statistical significance increases (p = 0.0046). Several other removal ranges were tested using the chi-square test and it has been found that the removal range of 55–80% generates the most significant result. For example, if the removal range is further expanded to be 50–85%, the significance drops (p = 0.037). Unless otherwise stated, the rest of our results were obtained with a removal range of 55–80%.

To obtain 7 points for a shRNA, it must pass multiple phase II filters. What filters/rules contribute to the statistical bias of the 7-points-cutoff? After analyzing all the design rules, we found that the shRNAs scoring no less than 7 are perfectly correlated with the f-dga filter (R = 1.0). The f-dga filter in phase II is defined such that any shRNA whose free energy (ΔG) of the 5-mer at 3' terminal is no less than -3.2 would gain 1 point. The free energy of the 5-mer at 3' terminal, ΔG_as-5 _(Subscript _as _in ΔG_as-5 _represents the 3' terminal while _ss _represents the 5' terminal, _-5 _means the first 5 nucleotides), shows some bias between functional and nonfunctional shRNAs. Functional shRNAs prefer the ΔG_as-5 _to be no less than -3.2 while disfavoring ΔG_as-5 _being less than -3.2 (p = 0.0046). This shows that the statistical bias behind the 7-points-cutoff is due to the ΔG_as-5 _values of shRNAs, and suggests that having ΔG_as-5 _larger than -3.2 increases the probability obtaining functional shRNAs at the expense of missing some functional ones (shown in Table 1).

**Table 1:** Higher ΔG_as-5 _increases the probability that shRNAs are functional.

shRNA ΔG_as-5_		Number	Functional Rate
>= -3.2	Functional	155	81.6% (155/190)
		
	Not functional	35	

<-3.2	Functional	66	66.7% (66/99)
		
	Not functional	33	

Table 1 indicates that the enforcement of filter f-dga in the selection algorithm could improve the design success rate significantly at the expense of missing some functional shRNAs. The results in Table 1 were obtained using a removal range of 55–80%. Even with no removal range, enforcing the filter f-dga significantly improves the shRNA design success rate to 76.0% (222 out of 292). This suggests that the high energy state at the 3' terminal has positive impact on shRNA efficacy.

The free energy of 3' terminal is computed with the first 5 terminal base pairs as suggested by Ui-Tei and Levenkova [12,25]. It is reasonable to ask if other base pair lengths could better represent the free energy of the 3' terminal. The free energies of different base pair lengths (2 to 10) from both the 5' terminal and 3' terminal are computed and chi-square test for independence is conducted to evaluate if there is any significant correlation between the free energy values and the shRNA efficacies. It is found that the free energy values of the first 6, 7 or 8 base pairs from 3' terminal have statistically significant correlation with the shRNA efficacy (p values are 0.0046, 0.0005 and 0.0011 respectively). Based on the p values, it looks like that ΔG_as-7 _is a better representation of the free energy than ΔG_as-5_. Also it is found that ΔG_ss-6 _and ΔG_ss-7 _show significant correlation with shRNA efficacy (p values are 0.00447 and 0.00546 respectively). Making judgment from the p values, it is not difficult to observe that the high energy state at the 3' terminal (5' end of antisense strand) can better differentiate functional and nonfunctional shRNAs than the high energy state at the 5' terminal (5' end of sense strand) can. This confirms the strand bias discovered before [14,15]. However, it turns out that it is more promising to use either ΔG_as-7_, ΔG_ss-6 _or both to predict the efficacy of shRNAs. The above results are summarized in Table 2. Our discovery differs from the recent findings on siRNA made by Matveeva and Shabalina [19,20]. Matveeva and Shabalina found that the ΔG of the first and last two base pairs at the two terminals correlates well with siRNA efficacy. The difference between our results and theirs might be due to the fact that different data sets were used or to the differences between chemically synthesized siRNAs and shRNAs.

**Table 2:** Using either ΔG_as-7_, ΔG_ss-6 _or both can better predict the efficacy of shRNAs.

Energy profile	ΔG_as-5_	ΔG_as-7_	ΔG_ss-6_	ΔG_as-7 _or ΔG_ss-6_or both
Energy criteria	>= -3.2	>= -6.6	>= -7.0	
P value of Chi-test	0.0046	0.0005	0.0045	0.0000001
True positive	155	155	115	192
False positive	35	32	22	39
True negative	33	36	46	29
False negative	66	66	106	29
ROC specificity	0.49	0.53	0.68	0.43
ROC sensitivity	0.70	0.70	0.52	0.87

ROC: Receiver Opearting Characteristic. True positives (TP) are those shRNAs which are experimentally tested to be functional and they meet the energy criteria. False positives (FP) are those shRNAs which meet the energy criteria but are experimentally tested to be nonfunctional. True negatives (TN) are those which are nonfunctional in experiments and fail the energy criteria. False negatives (FN) are those which fail the energy criteria but are functional experimentally. ROC specificity = TN/(TN+FP); ROC sensitivity = TP/(TP+FN).

Without any removal range, all the shRNAs whose ΔG_as-7 _>= -6.6 or ΔG_ss-6 _>= -7.0 or both have averaged suppression efficacy of 76.98%, while those shRNAs with no high energy state at either end have averaged suppression efficacy of 65.83%. A two-sample t-test reveals that the difference between the two averaged efficacy is very significant (p = 0.0000047). This confirms that the use of either ΔG_as-7_, ΔG_ss-6_or both could significantly improve the selection of functional shRNAs.

Recent research has shown that the siRNA sequence characteristics could be helpful in predicting siRNA efficacy [16-20]. To test this, we compiled a set of 404 sequence characteristics parameters and used linear least square analysis (multiple regression analysis) in an effort to identify the most influential sequence characteristics. Since the two terminals' energy profiles contribute to shRNA efficacy, the least square analysis includes the two energy parameters. The 406 parameters are generated as follows:

• The free energy of the first 7 base pairs of the antisense string (if >= -6.6, then value 1; otherwise, value -1).

• The free energy of the first 6 base pairs of the sense string (if >= -7.0, value equals 1; otherwise value equals -1).

• At each of the 21 positions, the nucleotide could be either A, C, G, T. For the presence of each nucleotide, there are 4 values generated. For example, for A, the 4 parameters are 1 0 0 0 while for G they are 0 0 1 0. So there are 84 parameters for the 21 nucleotides.

• For each pair of nearest neighbors, there are 4 × 4 = 16 parameters. For example, for AC, they are 0 1 0 0 0 0 0 0 0 0 0 0 0 0 0 0. So there are 16 × 20 = 320 parameters.

For this analysis, the removal range of 60–75% was selected since it provides a larger sample size than the removal range 55–80%. For each round of linear square analysis, three-fourth of shRNAs from the shRNA dataset were randomly selected as the experimental group to identify the best set of coefficients for the 406 parameters, while the remaining one-fourth of the samples were used to evaluate how well the parameters and the found coefficients can predict the shRNA efficacy. The experiment was repeated 8 times. The averaged prediction accuracy is about 68.55%, which cannot match the prediction by only using the energy profiles. Nevertheless, the linear analysis revealed that for the 8 repetitions of the experiments, some parameters always showed significant positive impact on the shRNA efficacy while others always had significant negative influence. These parameters are listed in Table 3.

**Table 3:** Sequence characteristics that consistently show either positive or negative impacts on shRNA efficacy in Least Square Analysis.

Positive Parameters		
Parameter	Position	Coefficient

G	3	8.01
G	5	9.11
A	11	7.59
T	14	6.16
CA	6–7	11.48
CC	8–9	11.63
TG	10–11	19.58
GA	13–14	31.32
TA	17–18	6.36
TA	18–19	24.11
Negative Parameters		
Parameter	Position	Coefficient
TA	4–5	-35.04
TA	6–7	-23.35
GG	8–9	-21.00
GC	9–10	-24.66
GA	17–18	-15.08

Different parameter sets are also compiled in search for the best parameter set. For example, some parameter sets exclude energy profiles and some include GC ratio, etc. It is worthy to note that with different parameter sets, the coefficients change dramatically while the predication accuracy does not improve. This makes us wonder whether the findings by linear least square analysis are nuisances. However, we did notice that on average, the nucleotide pairs have larger impact than single nucleotides. For example, in Table 3, the absolute average value of the coefficients of pairs is 20.33 while that of the single nucleotide is 7.73. The T-test shows that the p-value of the difference is 0.00034. This might indicate that longer sequence characteristics affect shRNA efficacy more than shorter sequence characteristics.

As there are reports that local RNA target structure influences siRNA efficacy [26,27], we then wanted to know if this could happen to shRNA. The secondary structure of mRNA could be formed for various reasons, but internal palindromes and repeated sequences are two causes in some cases. Please be aware that here we only performed the analysis statistically. The result does not indicate how much the secondary structure could impact the shRNA efficacy.

Initially we considered all possible palindromes and repeated sequences of length 7 or more that could involve any part of the 21 shRNA nucleotides. No significant results were found. We then only considered the possible palindromes and repeated sequences of length 8 or more that could only involve any part of the 6 base pairs from 5' terminal or of the 7 base pairs from 3' terminal. It was found that the number of possible palindromes of length 8 or more involving 3' terminal shows statistical bias between functional and nonfunctional shRNAs. Nonfunctional shRNAs tend to have more possible palindromes. This bias is most significant with palindromes of length 9 or more involving any part of the 7 base pairs from the 3' terminal. By Chi-Square test, the statistical significance is p = 0.011 with removal range of 55–80% and p = 0.0001 with removal range of 60–75%. Please notice that here we assumed that more possible palindromes implies higher probabilities for the terminal to be involved in secondary structure formation. If the assumption is valid, then the above result implies that secondary structure involving the 3' terminal could negatively impact the shRNA efficacy.

The above experiment targets the 7 base pairs at the 3' terminal. It is reasonable again to ask if other lengths of nucleotide sequences at 3' terminal will show similar statistical bias. Unsurprisingly, we found that all 3' terminal sequences of lengths 1 to 7 show similar statistical bias, i.e. functional shRNAs tend to have less possibility for all the terminal sequences of lengths 1 – 7 to be involved in palindrome formation. The most statistical bias is found with terminal sequence of length 6 (p < 0.000004 with removal range of 60–75%, p = 0.0006 with removal range 55–80%). This discovery motivates us to combine it with energy profile in order to further improve the efficacy predication. Our investigation has yet to show that this statistical bias could further improve the predication accuracy. This is not surprising since the possible palindrome structure could affect the terminal energy state. The two variables, the terminal energy state and the possible secondary structure are interfering with each other. A multivariate statistical analysis or recursive partition approach might help bring more lights into our future investigation.

## Conclusion

The default setting of the three-phase algorithm is relatively stringent. Under this default setting, the algorithm cannot design shRNA sequences for approximately 8% of genes from human Refseq database [23]. It is possible that many functional shRNA sequences are missed by this algorithm. However, when there could be enough shRNA sequences designed for a given gene, this algorithm with the default stringent setting promises a good probability for functional shRNAs.

It has been confirmed by several studies that the free energy profile at the two terminals is the most critical factor relating to siRNA efficacy [7,15-20]. Our analysis on shRNA dataset confirms that this belief applies to shRNA design. However, there is difference. For chemically synthesized siRNA, it was first found that the high free energy of first 5 base pairs at each terminal correlated well with siRNA efficacy [12,25], and later other researchers discovered that it might be the high free energy of the first 2 base pairs [19,20]. Our results show that shRNA efficacy can be predicted more effectively using the free energy profile of the first 6 base pairs at the 5' terminal and the first 7 base pairs at the 3' terminal. Currently we are not clear about the reason behind the difference, though the difference might be due to the differences between chemically synthesized siRNA and shRNA.

Internal palindrome is one of several causes that help RNA molecules form secondary structures. Our analysis found that shRNAs with more possible palindromes involving the 3' terminal tend to affect shRNA efficacy negatively, especially those possible palindromes that are of length 9 or more and involve the 7 terminal nucleotides. RNA secondary structure involving the terminal could limit the accessibility of the terminal, which might explain why the secondary structure could negatively impact shRNA efficacy. However, our result is very primitive since it is obtained with possible palindromes only and is only a statistical analysis result. Our future work will make use of software mfold to more precisely elucidate the relationship between shRNA efficacy and RNA secondary structure. If more positive relationships are found, confirmation by biological experiments will follow.

## Methods

### Cell culture and shRNA delivery

293H cells (Invitrogen) were cultured in D-MEM supplemented with 10% FBS and 1× non-essential amino acids (Invitrogen) for no more than 15 passages. Gene specific shRNA sequences were designed using the three-phase algorithm [23]. Negative control is a shRNA that has a random sequence and no homology to the mammalian transcriptome. All shRNAs were cloned into the pGeneClip™ hMGFP vector (Promega) to generate SABioscience SureSilencing™ shRNA plasmids. Transfection grade SureSilencing™ plasmid (0.8 mg) mixed with Lipofectamine 2000 reagent (Invitrogen, 3 mL) was delivered to 80,000 cells in a 24-well plate format. Culture media were changed 24 hours after transfection. Transfection efficiency was estimated by following the expression of GFP using fluorescence microscopy. After 48 hours, total RNA was extracted using the ArrayGrade™ Total RNA Isolation Kit with gDNA cleanup by TURBO DNase™ (Ambion).

### Real-time RT-PCR

cDNA was synthesized from total RNA using the ReactionReady™ First Strand cDNA Synthesis Kit. Real-time PCR was performed using RT2 SYBR Green qPCR Master Mixes on the Bio-Rad iCycler real-time PCR system or the Stratagene Mx3000 realtime PCR system.

### Gene knockdown efficiency calculation

Detailed description of knockdown success rate and its calculation is given in [28]. Real-time RT-PCR method was chosen to measure the relative mRNA levels between cells (293H from Invitrogen) transfected with a negative control shRNA and the cells transfected with target-specific shRNA. The percentage knockdown of a gene was calculated using the ΔΔCt method [29]. Basically, the expression level of our gene of interest is normalized to an internal control gene (GAPDH here) to obtain the ΔCt value, before it is compared between two differentially treated cells (thus the ΔΔCt). Since this validation process is complex, involving many different steps, we have built a statistical model to capture all the variations in the whole process [28]. By this model, we can mature the 95% confidence interval (CI) of the percentage knockdowns of any given shRNA. So our definition of a "successful" design is the shRNA that has a mean knockdown (KD) above 70%, with a lower bound of KD above 55.5% (with 95% CI). Please notice that all experiments were repeated for three times, and only those experiments with transfection efficiency greater than 80% and good PCR replicate consistency were included for analysis.

## Competing interests

The authors declare that they have no competing interests.

## Authors' contributions

HZ carried out the statistical analysis of the data and drafted the manuscript. XZ participated in the biological data preparation and revised this manuscript critically. All authors read and approved the final manuscript.
